# Multifunctional Polymer Memory via Bi‐Interfacial Topography for Pressure Perception Recognition

**DOI:** 10.1002/advs.201902864

**Published:** 2020-02-25

**Authors:** Xiangjing Wang, Zhe Zhou, Chaoyi Ban, Zepu Zhang, Shang Ju, Xiao Huang, Huiwu Mao, Qing Chang, Yuhang Yin, Mengya Song, Shuai Cheng, Yamei Ding, Zhengdong Liu, Ruolin Ju, Linghai Xie, Feng Miao, Juqing Liu, Wei Huang

**Affiliations:** ^1^ Key Laboratory of Flexible Electronics (KLOFE) & Institute of Advanced Materials (IAM) Nanjing Tech University (NanjingTech) 30 South Puzhu Road Nanjing 211816 China; ^2^ Key Laboratory for Organic Electronics and Information Displays & Institute of Advanced Materials (IAM) Nanjing University of Posts & Telecommunications (NUPT) 9 Wenyuan Road Nanjing 210023 China; ^3^ National Laboratory of Solid State Microstructures School of Physics Collaborative Innovation Center of Advanced Microstructures Nanjing University Nanjing 210093 China; ^4^ Shaanxi Institute of Flexible Electronics (SIFE) Northwestern Polytechnical University (NPU) 127 West Youyi Road Xi'an 710072 China

**Keywords:** memristors, multimode, nanoholes, nanowrinkles, recognition memory

## Abstract

Emerging memory devices, that can provide programmable information recording with tunable resistive switching under external stimuli, hold great potential for applications in data storage, logic circuits, and artificial synapses. Realization of multifunctional manipulation within individual memory devices is particularly important in the More‐than‐Moore era, yet remains a challenge. Here, both rewritable and nonerasable memory are demonstrated in a single stimuli‐responsive polymer diode, based on a nanohole‐nanowrinkle bi‐interfacial structure. Such synergic nanostructure is constructed from interfacing a nanowrinkled bottom graphene electrode and top polymer matrix with nanoholes; and it can be easily prepared by spin coating, which is a low‐cost and high‐yield production method. Furthermore, the resulting device, with ternary and low‐power operation under varied external stimuli, can enable both reversible and irreversible biomimetic pressure recognition memories using a device‐to‐system framework. This work offers both a general guideline to fabricate multifunctional memory devices via interfacial nanostructure engineering and a smart information storage basis for future artificial intelligence.

## Graphical abstract

A general guideline for fabricating a multimode memristor based on spontaneously formed bi‐interfacial nanostructures is proposed to simulate the somatosensory recognition memory.

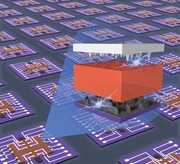

The smart memory system in the human brain is based on recognition memory, sensory memory, and high‐density storage with ultralow power consumption.^1^, ^2^, ^3^ In particular, the episodic memory, that combines both sensory and recognition memory, allows an individual to store and recall a specific and an important event taking place at a particular time and/or place for long period of time; while ordinary events only for relatively shorter period of time.^4^, ^5^, ^6^ Inspired by this, an ideal artificial memory cell is expected to perform multimode, multilevel, and low energy operations in response to external or internal stimuli.^7^, ^8^ Stimuli‐responsive resistive switching (RS) in two‐terminal devices comprising of inorganic oxides, organic molecules, polymers, carbon nanomaterials, and their composites have emerged as attractive candidates for future memory materials.^9^, ^10^, ^11^, ^12^, ^13^, ^14^ Among these systems, polymer memories are especially promising, owing to their tunable structures, tailorable properties, facile engineering, and solution‐phased fabrication processes.^15^, ^16^, ^17^, ^18^, ^19^ Despite the great progress made in polymer memories for data storage and artificial synapse,^20^, ^21^, ^22^, ^23^, ^24^ most of these systems could only perform single function. Therefore, it is not possible to fulfill several simultaneous requirements for practical memories, such as multimode function, varying speed, multilevel storage, and low power consumption.

It has been previously shown, both experimentally and theoretically, that the memory performance of a polymer diode depends particularly on its active composition, device structure, interfacial design, and external stimuli.^25^, ^26^, ^27^, ^28^, ^29^, ^30^ Several strategies, including mechanism synergy, charge trap modulation, multilayer stacking, interfacial nanostructure, component blending, and suppressed stimuli, have been employed to boost and diversify the memory function of a single cell, resulting in multilevel storage, rectifying memory, low energy operation, and volatile and nonvolatile hybrid materials based memory.^31^, ^32^, ^33^, ^34^, ^35^, ^36^, ^37^ However, to establish a step‐by‐step guide of constructing multifunctional memory, to simultaneously achieve multimode, multilevel storage, and low power consumption, is necessary. Here, we report the fabrication of vertical polymer memory diodes, based on spontaneously formed bi‐interfacial nanostructures, which exhibit stimuli‐responsive multifunctions; and at the same, we demonstrate pressure recognition and memory efficacy by using a device‐to‐system level simulation framework.

**Figure** **1**a depicts schematically the device prototype: namely, a semiconductive poly[2‐methoxy‐5‐(2‐ethylhexyloxy)‐1,4‐phenylenevinylene] layer with uniformly distributed nanoholes (nh‐MEH‐PPV) is sandwiched between metallic aluminum (Al) electrode and metal‐like nanowrinkled reduced graphene oxide (nw‐rGO) electrode. The topographic atomic force microscopy (AFM) image reveals rather uneven surface of bottom nw‐rGO electrode with nanowrinkle height of ≈16 nm, as can be noticed in the left and right bottom parts of Figure 1b and the bottom part of Figure 1c. It is well‐known that nanowrinkles play a crucial role in write‐once‐read‐many times (WORM) memory, owing to the enhancement of localized field emission induced by the atomic thin edges of the nanowrinkles.^27^ To incorporate a nonvolatile repeatable switching mode (so‐called flash mode) into the same cell and, simultaneously, to diminish the effect of the random nature of the polymer memory (as the formation and the rupture of a conductive filament (CF) are two stages of a stochastic process that often occurs at the weakest locations of RS medium layer),^38^ a semiconductive nh‐MEH‐PPV layer with uniformly distributed nanoholes was deposited via breath‐figure method (Figure S1, Supporting Information).^39^ In the topographic AFM image shown in the left and right top of Figure 1b, an ensemble of circular holes, with diameters ranging from 110 to 150 nm (results summarized in the left of Figure 1d), occupy the top surface of the MEH‐PPV film. These nanoscale holes show pin‐like morphology with a typical depth of ≈10 nm (results summarized in the right of Figure 1d). Their tips point toward the nw‐rGO electrode (in the top of Figure 1c); thus they do not penetrate in the whole nh‐MEH‐PPV film. Then, by subsequent thermal deposition, Al atoms could migrate into the nanoholes and form a weaker location with respect to the surrounding area. The pin‐enhanced local electrical fields could facilitate the ionization of Al atoms, accelerate the migration of Al^3+^ ions, and simultaneously control the location of CFs under external stimuli.^40^

**Figure 1:**
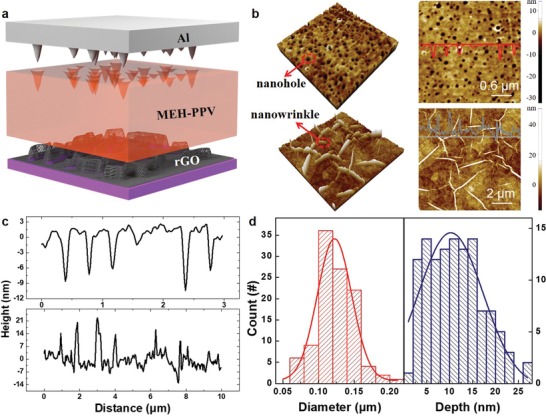
Device structure and characterization of nanohole‐nanowrinkle interface. a) Schematic of a bi‐interfacial engineered memory with an intervening nh‐MEH‐PPV film sandwiched between bottom nw‐rGO and top Al electrode. b) AFM image of the topography of intervening nh‐MEH‐PPV film with uniformly distributed nanoholes (top left and top right) and bottom nw‐rGO electrode with nanowrinkles (bottom left and bottom right), respectively. The red line in top right and gray line in bottom right represent the height profiles across nh‐MEH‐PPV film and nw‐rGO electrode, respectively. c) Height profiles indicate that the nanoholes on top of the nh‐MEH‐PPV film feature pin shape (typical depth of ≈10 nm) toward nw‐rGO (top); while the peak of nanowrinkles toward Al (bottom) has typical height of ≈16 nm; therefore they do not penetrate into the whole nh‐MEH‐PPV film. d) Statistical histogram of the diameter and depth of nanoholes, as measured by AFM and fitted by a Gaussian function (red line for diameter and blue line for depth).

Next, the switching performance of the device with a bias applied to the top Al electrode and bottom nw‐rGO electrode grounded is investigated. The device has a rectifying behavior with a high rectification ratio and excellent stability, as well as promising cell‐to‐cell variation at low bias (−2 to 2 V and −3 to 3 V) (**Figure** **2**a; Figure S2, Supporting Information). This indicates ideal Schottky contacts formed at the nw‐rGO/nh‐MEH‐PPV/Al interfaces, in accordance with the energy level diagram (Figure S2, Supporting Information) and consistent with the AFM results. Aside from the rectifying mode, stable flash mode and WORM mode are combined in individual cells via precise tuning of the diverse bias (Figure 2a). The flash mode is mainly operated at the voltage range from −4 to 4 V with great cycle‐to‐cycle variability (**Figure** **3**a), outstanding retention (Figure S3, Supporting Information) and localized set voltage of ≈2.7 V as well as reset voltage of ≈−2.3 V (in the top of Figure 3d). With a further increased positive voltage, another electrical transition (mainly taking place at 4.15 V) from the low resistance state (LRS 1) to a lower resistance state (LRS 2) is observed (Figure 2a). The cell maintained in the LRS 2 in the subsequent sweep under larger negative voltages (−6 to 6 V or −7 to 7 V) and absence of external power, suggests a nonerasable memory behavior with a superior retention of 10^4^ s and a high ON/OFF ratio of ≈10^4^ (Figure S4, Supporting Information). Note that, ultralow power dissipation can be achieved continuously by modulating current compliance (CC), with no obvious degradation for each electrical property (Figures 2b and 3b). Impressively, the two diverse LRSs, as well as the high resistance state (HRS), enable the realization of a ternary memory (Figure 2c; Figure S5, Supporting Information), indicating high density data storage. The set and reset voltages for flash mode exhibit negligible fluctuation; while the set voltage in the WORM mode obviously increase to ≈7 V (with a CC of 10^−5^ A) and ≈9 V (with a CC of 10^−6^ A), respectively (Figure 3a,d, Figure S6, Supporting Information). A possible explanation of this observation is that the carriers, which are accumulated around the peaks of nanowrinkles, are not sufficient to form a strong field with a CC; thus a higher set voltage is required.^27^ Also, pulsed bias with gradually increased amplitude and progressively broadened span are utilized to test the switching speed of the memory device. The device exhibits a flash switching within 0.1 s under the applied set pulse of 4 V in amplitude. Meanwhile, the minimum pulse width, to realize WORM switching, decreases with increasing pulse voltage amplitude; that is, ≈1 ms for an amplitude of 5 V and only ≈10 µs for an amplitude of 10 V, as shown in Figure 3e, during several switching cycles (see Figure 3f).

**Figure 2:**
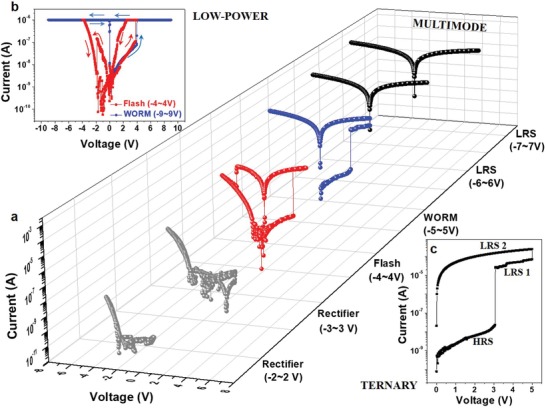
Multimode memristive characteristics and capabilities of low‐power dissipation and ternary memory of the device. a) DC switching curves under various bias regime of the device. At low bias regime of −2 to 2 and −3 to 3 V, the device shows no switching but a rectifying behavior (gray line). As the bias applied to two terminals exceeds 3 V, a nonvolatile rewritable switching behavior (flash mode) is observed (red line) with an abrupt decrease in the resistance from the HRS to an LRS 1. With a further increased bias, another transition from LRS 1 to LRS 2 takes place at ≈5 V (blue line). The LRS 2 is maintained during the successive sweeps with larger bias (black line) and in the absence of external power (labelled as WORM mode). b) Typical *I*–*V* curves of a device with a CC of 10^−6^ A, where the arrows indicate the sweep directions (red arrows for flash mode and black arrows for WORM mode). Basic functions are maintained in the presence of current limitation, suggesting the power consumption can be cut down since it depends on the LRS current. c) *I*–*V* characteristics of a device without CC and distinctly diverse resistance states are developed, allowing ternary memory (LRS 1 for flash mode and LRS 2 for WORM mode).

**Figure 3:**
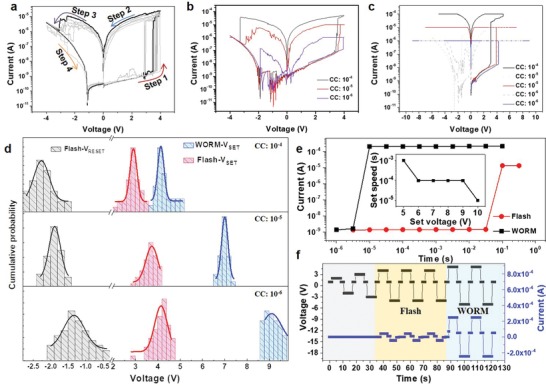
Electrical characterization of the device. a) *I*–*V* curves of the device under flash mode (black line for the first switching and gray lines for reversible switching). b) *I*–*V* curves of the device with varying CC for flash mode (black line for 10^−4^ A, red line for 10^−5^ A, and blue line for 10^−6^ A, respectively). c) *I*–*V* curves of the device with varying CC for WORM mode are shown (black line for 10^−4^ A, red line for 10^−5^ A, and blue line for 10^−6^ A, respectively). Grey dashed (and dotted) line represents the *I*–*V* curve in which the device remains in flash mode until an applied threshold bias of 5, 7, and 9 V (Figure S6, Supporting Information, the second transition from flash mode to WORM cannot be observed due to current limitation). d) Statistical histogram of set voltage for flash mode (red), WORM mode (blue), and reset voltage for flash mode (gray) without current limitation (top) and with CC of 10^−4^ A (top), 10^−5^ A (mid), and 10^−6^ A (bottom), respectively. The Gaussian fits demonstrate negligible fluctuation of set (≈2.90 V) and reset voltage (≈−2.25 V) for flash mode, due to current limitation; while there is a clear increase in set voltage (≈7 and 9 V) for WORM mode. e) Switching speed measurement of a device is shown. The bias applied to the device is 4 V for the set of flash mode and the device can be switched within 0.1 s (red line). The switching time of WORM mode is inversely proportional to the applied bias (inset) and it has a minimum value of ≈10 µs under maximum value of 10 V bias. f) switching cycles of a device measured with gradually increased voltage pulses are shown (black curves are the applied bias and the blue curves are the related currents).

To investigate the role of the nanohole‐nanowrinkle interfacial structure in the memory performance of the device as interface is essential to the switching behaviors,^41^ several reference devices with diverse bi‐interfacial structures (e.g., smooth‐nanowrinkle, nanohole‐smooth, and smooth‐smooth structures) are fabricated. Both the smooth‐nanowrinkle and nanohole‐smooth devices exhibit a similar rectifying behavior which undergoes at lower bias compared to the nanohole‐nanowrinkle device (Figures S7 and S9, Supporting Information), which indicates both homogeneity of the hole or wrinkle nanostructures and the high quality conductor–semiconductor contacts. However, in terms of memory behavior, their *I*–*V* curves show only flash mode in the nanohole‐smooth structure (Figures S7 and S8, Supporting Information), only WORM mode in the smooth‐nanowrinkle structure (Figure S9, Supporting Information), and absence of any type of memory effect in the double‐smooth structure (Figure S10, Supporting Information), while undergoing the identical stepwise sweep of −4 to 4, −5 to 5, and −6 to 6 V, respectively. These observations justify the hypothesis that the flash mode is triggered by the nanoholes on the surface of the MEH‐PPV medium layer; whereas the WORM is induced by the nanowrinkles of the rGO electrode. Impressively, the reference devices with different MEH‐PPV film thicknesses show that a reasonable thickness (e.g., 50 nm) is critical for achieving multimode switching behavior, as only LRS, WORM, and HRS are achieved for the thicknesses of 10, 80, and 190 nm, respectively (Figure S11, Supporting Information).

In order to demonstrate the universality of our bi‐interfacial engineering strategy, switching behavior of other polymer‐based bi‐interfacial devices, such as those based on poly(3‐hexylthiophene‐2,5‐diyl) (P3HT) and insulating polymethyl methacrylate (PMMA), is elaborated (Figures S12 and S13, Supporting Information). By varying the concentration and spin‐casting speed of the polymers, the *I*–*V* curves of the optimized devices exhibit similar multimode switching behavior compared to the bi‐interfacial system based on MEH‐PPV mentioned above (Figures S12 and S13, Supporting Information), i.e., flash mode at relatively lower bias and WORM mode at higher bias. This strongly suggests that the multifunctional memory performance of polymer diodes is based on the wrinkle‐hole bi‐interfacial nanostructure.

In addition, for the multimode memories taking place in a single cell at selectable working regimes, the nature of resistance switching should also be different in accordance with the various writing speeds. For flash mode, the logarithmic and linear fittings of the *I*–*V* curves for the positive sweep region suggest ohmic conduction in the LRS with a slope of ≈1. On the other hand, thermionic emission (TE) (ln(*V) ∝ V* ^1/2^) with concomitant space‐charge limited conduction (SCLC) (*I ∝ V* ^2^) dominates the electrical conduction in the HRS (**Figure** **4**a). To identify the transport behavior of the resistive switching, temperature dependence of the device current needs to be examined. As shown in Figure 4b, the current in the HRS shows a positive response to temperature, suggesting a typical thermally activated transport behavior. The current in the LRS is inversely proportional to temperature, which is indicative of a metallic filament mechanism. To verify this mechanism, the dependence of the cell resistance on area is further studied. As shown in Figure 4c, as the diameter of circular‐shaped electrode increases from 64 to 480 µm; there is a considerable decrease of the HRS resistance from 9.5 × 10^8^ to 9.4 × 10^7^ Ω, which can be attributed to the homogeneous current flow across the whole cell. In contrast, the LRS resistance depends much less on the electrode diameter; there is only a slight decrease from 1.6 × 10^5^ to 1.1 × 10^5^ Ω, which confirms the metallic conduction behavior. As a result, the switching mechanism of flash mode can be understood as formation and rupture of metal Al filaments (Figure 4d; Figure S15, Supporting Information). In contrary, the switching mechanism of WORM is distinct in accordance with the unusual fitting curves and variable switching speed, as seen in Figure 4e and Figure 3e, respectively. In addition to the two similar portions of thermionic emission and SCLC, a charge tunneling process (*I ∝ V*) is observed in the WORM regime (Figure 4e). Such a tunneling process has been known to often cause resistive switching in polymer memory devices based on interfacial nanostructures, with nonreversible formation of carbon‐rich filament derived from dehydrogenation of polymer under local enhanced electric field.^27^ Therefore, the second transition and an LRS 2 with a higher current density in the WORM mode indicates that the CFs consist of both the nanohole‐induced intermittent metal filament (low voltage regime) and the nanowrinkle‐induced carbon‐rich filament (high voltage regime) rather than a single continuous metallic filament (Figure 4h; Figure S15, Supporting Information).

**Figure 4:**
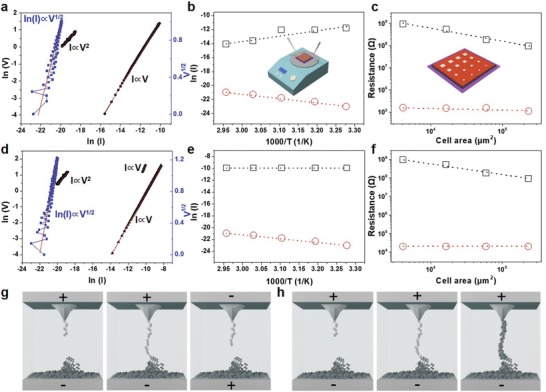
Charge conduction and resistive switching mechanism. a) ln (*I*)–ln (*V*) relationship for the forward bias regime of flash mode is shown. The fitting results indicate that thermionic emission (ln(*V*) vs *V* ^1/2^, blue line) and space‐charge limited conduction (SCLC, *I* ∝ *V* ^2^, black line in left) dominate electrical conduction in the HRS and there is ohmic conduction in the LRS 1 (black line in right). b) The temperature dependence of current in the HRS and the LRS 1; and c) the cell area dependence of resistance in the HRS and the LRS 1 are shown. d) ln (*I*)–ln (*V*) relationship for the forward bias regime of WORM mode is shown. The fitting results indicate that thermionic emission (ln(*V*) vs *V* ^1/2^, blue line) and space‐charge limited conduction (SCLC, *I* ∝ *V* ^2^, black line in left) dominate electrical conduction in the HRS and there is ohmic conduction in the LRS 1 and the LRS 2 (black line in right). e) The temperature dependence of current in the HRS and the LRS 1; and f) the cell area dependence of resistance in the HRS and the LRS 1 are shown. g,h) Schematic illustrations to propose a resistive switching model for the multifunctional device. In the case of flash mode (g), the switching behavior is ascribed to the formation and rupture of the Al filaments; in the case of WORM mode, the switching behavior results from the localized electrical field induced formation of carbon‐rich filaments.

To mimic a smart episodic memory,^7^ a pressure recognition memory system (PRMS), that is able to respond to diverse external stimuli with varying intensities, is introduced. This PRMS has two components: a resistive pressure sensor acting as a receptor to convert pressure stimuli into electrical signals and a multifunctional memory serving as a memory neuron, as depicted in **Figure** **5**a. The sensor exhibits a gradually decreasing resistance with a progressively increasing applied pressure. Such a reduction in resistance, in turn, results in an increase of partial voltage on the tandem memory unit; consequently two distinct resistive switching behavior of the memory are in effect. As a proof‐of‐concept demonstration, memory arrays comprising of 4 × 5 bits are fabricated and connected to the sensor in series. As an essential prerequisite, the sensor is tested for stable performance, in terms of working range, reproducibility, and uniformity. No obvious degradation during the dynamic response to externally applied pressure of ≈1 g and ≈5 g, as shown in Figure 5b,c, respectively, are observed. Initially, both the pressure sensor and the memory are at the HRS, where the original resistance of the pressure sensor is larger than that of the memory; thus the partial voltage acquired for the memory could not reach *V*_set1_ (set voltage for flash mode) and failed to activate the device to the LRS from the HRS. As the applied pressure exceeds 1 g, yet less than 5 g (referred as “touch”), the minimum resistance of the pressure sensor is around 10^6^ Ω, which is comparable to that of the HRS of the tandem memory. Accordingly, the partial voltage on tandem memory increases to *V*_set1_, but no more than *V*_set2_ (set voltage for WORM mode), indicating that a recent memory could be erased (or “forgotten”) by applying a reverse bias on the memory. On the other hand, as the pressure exceeds 5 g (referred as “hit”), the resistance of the pressure sensor (<10^4^ Ω) becomes several orders of magnitude lower than that of the HRS or even the LRS of the tandem memory. This suggests that the partial voltage could approach *V*_set2_ and consequently leads to the remote memory that could not be forgotten by either increasing the positive bias or applying a negative bias, as shown in Figure 5d,e, respectively. Figure 5f displays the information recorded in the memory arrays which form pixel maps by MATLAB software. The bit cells in the memory arrays are programmed into binary bits representing “IAM” (acronym for “Institute of Advanced Materials”), with both LRS 1 and LRS 2 representing logic “1” and HRS for logic “0.” Not only pressure sensors, but also other sensors (temperature, light, sound, and so on) could also be integrated in this memory system; therefore to mimic episodic memory by several external stimuli is within reach.

**Figure 5:**
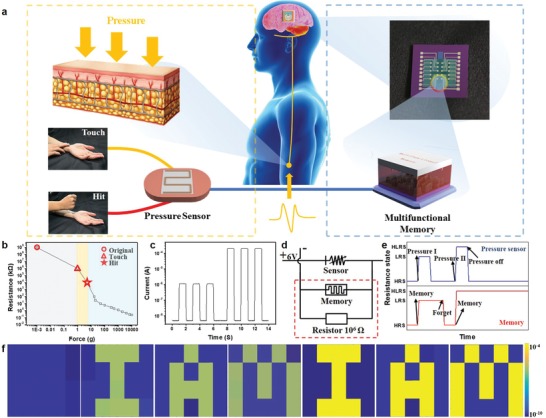
A pressure sensory and recognition memory (PSRM) system and its characterization. a) The artificial PSRM in comparison with a biological one is presented. Top: pressure with varying intensities, applied onto skin, can be recognized and memorized; and bottom: the bio‐inspired PSRM, which is a system acting as a pressure sensor and a memory device at the same time, are given. b) Resistance of a resistive pressure sensor and the related change in response to the change of applied pressure and c) response to applied pressure stimuli with two distinct intensities are plotted. d) A circuit diagram of the artificial PSRM and e) schematic illustration of the function of resistance states between pressure sensor and memory device are shown. In (d), the pressure sensor is connected to the memory device in series with a voltage supply. In (e), pressure with a relative low intensity reduces the resistance of the sensor; subsequently induces a voltage drop in the sensor and an increase of partial voltage in the tandem memory unit, which leads to a switching of the memory unit from the HRS to the LRS 1 (flash mode). The recorded information can be erased by a reverse bias. When the applied pressure overcomes the threshold, a further increase in voltage results in irreversible switching behavior and the memorized information becomes indelible. f) An example of information storage behavior of the PSRM for the applied pressure with varying intensities is described. The mappings are obtained by MATLAB program with current‐color transition.

In summary, a general guideline to fabricate polymer multimode memory devices is presented; both flash and WORM functions in individual cells via precise tuning of electrical stimuli are demonstrated. This is only possible by a device design, based on a solution‐processed bi‐interfacial hole‐wrinkle nanostructure, which does not require complex fabrication process, which is crucial for high yield and low‐cost manufacturing. The dual‐filament mechanism induced by the bi‐interfacial nanostructure also enables a ternary switching for high density data storage. Moreover, the power dissipation of device can be modulated by limiting the CC, suggesting high potential for ultralow power consumption electronics. Finally, the multimode memory, combined with a pressure recognition unit, demonstrates the capability of memorizing signals of “touch” and “hit” at different pressure levels, which is a significant progress toward emulation of human memory, especially for smart episodic memory.

## Experimental Section

##### Synthesis of Graphene Oxide (GO) Precursor

GO was synthesized by a modified Hummers method as follows: 1g expanded graphite and 0.5 g sodium nitrate and 23 mL sulfuric acid (98 wt%) were added into a 500 mL flask under ice water bath, followed by adding 3 g potassium permanganate slowly and kept stirring until forming a homogeneous solution. The solution was maintained at 35 °C and stirred for 8 h to avoid overheating and explosion. Then 46 mL deionized water with a temperature of 40 °C was added; then the flask was transferred into an oil bath and was slowly heated to 95 °C. A bright yellow solution was obtained after 20 min, which was followed by washing with hydrogen chloride (HCl) and deionized (DI) water; and subsequently the graphite oxide was obtained. By dispersing graphite oxide in mixed solution (DI water/methanol = 1/5), GO solution with diverse concentration was obtained.

##### Preparation of Nanowrinkled Reduced Graphene Oxide (nw‐rGO) Electrode

A Si wafer with a 300 nm SiO_2_ surface layer (SZJXTech, China) was used as substrate and ultra‐sonicated with DI water, ethanol, and isopropanol, each for 15 min, followed by drying with N_2_ flow and UV‐ozone treatment for 5 min. The substrate was then exposed to O_2_‐plasma for 5 min with 20 sccm O_2_ flow and 80 W rf power. The GO dispersion in mixed solution (0.5 mg mL^−1^ and DI water/methanol = 1/5) was spin‐coated on the precleaned SiO_2_ substrate, at 2500 rpm under ambient condition. The bottom nw‐rGO electrode was obtained by annealing the spin‐coated GO film at 1000 °C under Ar_2_/H_2_ gas mixture for 2 h. In the case of smooth reduced graphene oxide (s‐rGO) electrode, the GO precursor was ultra‐sonicated for 18 h before spin coating.

##### Preparation of Polymer Layers with and without Nanoholes

A poly[2‐methoxy‐5‐(2‐ethylhexyloxy)‐1,4‐phenylenevinylene] (MEH‐PPV, average *M*_w_ 40 000–70 000) layer with uniformly distributed nanoholes (nh‐MEH‐PPV) was obtained by spin coating the solution of MEH‐PPV in methanol (5 mg mL^−1^) on the obtained bottom nw‐rGO electrode at 1000 rpm for 40 s in damp air with humidity of 30–40%, followed by transfer in to an oven at 70 °C for 30 min to remove traces methanol. For the MEH‐PPV layer without nanoholes, the film was spin‐coated and annealed in a N_2_ filled glove box. The thickness of MEH‐PPV film ranged from 10 to190 nm was controlled by varying the spinning speed; and the typical thickness was 45 nm for all the experiments in this work (or otherwise as indicated).

##### Device Fabrication and Measurement

A 150 nm thick top Al electrode was thermally evaporated on the MEH‐PPV layer (with/without nanoholes) through a shadow mask with a diameter of 64, 128, 243, and 480 µm. Electrical characterization was performed by a Keithley 4200A‐SCS, in an ambient conditions.

##### Morphologies and Thickness Analysis

The rGO electrodes were spin casted on the precleaned and UV‐ozone treated SiO_2_ substrates, followed by thermal annealing. The MEH‐PPV films were spin casted on top of the rGO electrodes. The atomic force microscopy (AFM) images were obtained by using a Dimension 3100 (Veeco, CA) in tapping mode in ambient conditions. The thicknesses of the MEH‐PPV films were calculated from the AFM images.

##### Integration and Characterization of the Somatosensory Recognition Memory System

The pressure sensors (FSR 400) were purchased from SICHIRAY and utilized as received. The force applied onto it was measured with a force gauge. Resistance across the pressure sensors were measured by Keithley 4200A‐SCS. The polymer memory was connected to a resistor (≈10^6^ Ω) in parallel. Subsequently, the pressure sensor was connected to the polymer memory and the resistor in series. The electrical properties of the system were measured by a Keithley 4200A‐SCS in ambient conditions.

##### Data and Materials Availability

All data needed to evaluate the conclusions in the paper are present in the paper and/or the Supporting Information. Additional data related to this paper may be requested from the authors. All tests with volunteers were performed with the informed consent of the volunteers themselves.

## Conflict of Interest

The authors declare no conflict of interest.

## Supporting information

Supporting Information

## References

[1] A. K. Anderson , Y. Yamaguchi , W. Grabski , D. Lacka , Learn. Mem. 2006, 13, 711. 17101871 10.1101/lm.388906 PMC1783624

[2] M. W. Brown , J. P. Aggleton , Nat. Rev. Neurosci. 2001, 2, 51. 11253359 10.1038/35049064

[3] H. Tiitinen , P. May , K. Reinikainen , R. Näätänen , Nature 1994, 372, 90. 7969425 10.1038/372090a0

[4] T. Kitamura , S. K. Ogawa , D. S. Roy , T. Okuyama , M. D. Morrissey , L. M. Smith , R. L. Redondo , S. Tonegawa , Science 2017, 356, 73. 28386011 10.1126/science.aam6808 PMC5493329

[5] E. Tulving , F. Eustache , B. Desgranges , F. Viader , Rev. Neurol. 2004, 160, 9. 10.1016/s0035-3787(04)70944-3 15118553

[6] J. Poppenk , A. R. McIntosh , F. I. M. Craik , M. Moscovitch , J. Neurosci. 2010, 30, 4707. 20357121 10.1523/JNEUROSCI.5466-09.2010 PMC6632301

[7] Y. van de Burgt , A. Melianas , S. T. Keene , G. Malliaras , A. Salleo , Nat. Electron. 2018, 1, 386.

[8] D. Hassabis , D. Kumaran , C. Summerfield , M. Botvinick , Neuron 2017, 95, 245. 28728020 10.1016/j.neuron.2017.06.011

[9] S. Y. Xu , Q. Ma , H. Shen , V. Fatemi , S. Wu , T. R. Chang , G. Chang , A. M. M. Valdivia , C. K. Chan , Q. D. Gibson , J. Zhou , Z. Liu , K. Watanabe , T. Taniguchi , H. Lin , R. J. Cava , L. Fu , N. Gedik , P. Jarillo‐Herrero , Nat. Phys. 2018, 14, 900.

[10] W. Lin , S. Liu , T. Gong , Q. Zhao , W. Huang , Adv. Mater. 2014, 26, 570. 24339246 10.1002/adma.201302637

[11] S. Gao , X. Yi , J. Shang , G. Liu , R. Li , Chem. Soc. Rev. 2019, 48, 1531. 30398508 10.1039/c8cs00614h

[12] B. Cho , S. Song , Y. Ji , T.‐W. Kim , T. Lee , Adv. Funct. Mater. 2011, 21, 2806.

[13] E. C. Ahn , H.‐S. P. Wong , E. Pop , Nat. Rev. Mater. 2018, 3, 18009.

[14] M. Wang , S. Cai , C. Pan , C. Wang , X. Lian , Y. Zhuo , K. Xu , T. Cao , X. Pan , B. Wang , S. Liang , J. J. Yang , P. Wang , F. Miao , Nat. Electron. 2018, 1, 130.

[15] S. Han , L. Hu , X. Wang , Y. Zhou , Y. Zeng , S. Ruan , C. Pan , Z. Peng , Adv. Sci. 2017, 4, 1600435. 10.1002/advs.201600435 PMC5566243 28852609

[16] S. Goswami , A. J. Matula , S. P. Rath , S. Hedstrom , S. Saha , M. Annamalai , D. Sengupta , A. Patra , S. Ghosh , H. Jani , S. Sarkar , M. R. Motapothula , C. A. Nijhuis , J. Martin , S. Goswami , V. S. Batista , T. Venkatesan , Nat. Mater. 2017, 16, 1216. 29058729 10.1038/nmat5009

[17] Y. Ji , D. F. Zeigler , D. S. Lee , H. Choi , A. K. Jen , H. C. Ko , T. W. Kim , Nat. Commun. 2013, 4, 2707. 24176930 10.1038/ncomms3707

[18] G. Puebla‐Hellmann , K. Venkatesan , M. Mayor , E. Lortscher , Nature 2018, 559, 232. 29995866 10.1038/s41586-018-0275-z

[19] C. Liu , E. Franke , Y. Mignot , R. Xie , C. W. Yeung , J. Zhang , C. Chi , C. Zhang , R. Farrell , K. Lai , H. Tsai , N. Felix , D. Corliss , Nat. Electron. 2018, 1, 562.

[20] Y. Song , J. Jang , D. Yoo , S. H. Jung , S. Hong , J. K. Lee , T. Lee , Org. Electron. 2015, 17, 192.

[21] S. Salahuddin , K. Ni , S. Datta , Nat. Electron. 2018, 1, 442.

[22] P. Gkoupidenis , N. Schaefer , B. Garlan , G. G. Malliaras , Adv. Mater. 2015, 27, 7176. 26456708 10.1002/adma.201503674

[23] G. Milano , M. Luebben , Z. Ma , R. Dunin‐Borkowski , L. Boarino , C. F. Pirri , R. Waser , C. Ricciardi , I. Valov , Nat. Commun. 2018, 9, 5151. 30514894 10.1038/s41467-018-07330-7 PMC6279771

[24] Y. Lee , J. Y. Oh , W. Xu , O. Kim , T. R. Kim , J. Kang , Y. Kim , D. Son , J. B. H. Tok , M. J. Park , Z. Bao , T. W. Lee , Sci. Adv. 2018, 4, eaat7387. 30480091 10.1126/sciadv.aat7387 PMC6251720

[25] K. Krishnan , T. Tsuruoka , C. Mannequin , M. Aono , Adv. Mater. 2016, 28, 640. 26576756 10.1002/adma.201504202

[26] Z. Zhou , H. Mao , X. Wang , T. Sun , Q. Chang , Y. Chen , F. Xiu , Z. Liu , J. Liu , W. Huang , Nanoscale 2018, 10, 14824. 30043803 10.1039/c8nr04041a

[27] J. Chen , X. Wang , H. Lu , Z. Liu , F. Xiu , C. Ban , Z. Zhou , M. Song , S. Ju , Q. Chang , J. Liu , W. Huang , Small Methods 2018, 2, 1800048.

[28] J. Liu , Z. Yin , X. Cao , F. Zhao , L. Wang , W. Huang , H. Zhang , Adv. Mater. 2013, 25, 233. 23109141 10.1002/adma.201203349

[29] Q. Zhang , J. He , H. Zhuang , H. Li , N. Li , Q. Xu , D. Chen , J. Lu , Adv. Funct. Mater. 2016, 26, 146.

[30] S. Liu , P. Wang , Q. Zhao , H. Yang , J. Wong , H. Sun , X. Dong , W. Lin , W. Huang , Adv. Mater. 2012, 24, 2901. 22539455 10.1002/adma.201104307

[31] Y. Ma , H. Chen , F. Zhou , H. Li , H. Dong , Y. Li , Z. Hu , Q. Xu , J. Lu , Nanoscale 2015, 7, 7659. 25831970 10.1039/c5nr00871a

[32] H. Wang , B. Zhu , H. Wang , X. Ma , Y. Hao , X. Chen , Small 2016, 12, 3360. 27315137 10.1002/smll.201600893

[33] H. Tian , L. Zhao , X. Wang , Y. W. Yeh , N. Yao , B. P. Rand , T. L. Ren , ACS Nano 2017, 11, 12247. 29200259 10.1021/acsnano.7b05726

[34] Y. Shi , X. Liang , B. Yuan , V. Chen , H. Li , F. Hui , Z. Yu , F. Yuan , E. Pop , H. S. P. Wong , M. Lanza , Nat. Electron. 2018, 1, 458.

[35] X. Zhu , J. Lee , W. D. Lu , Adv. Mater. 2017, 29, 1700527. 10.1002/adma.201700527 28582597

[36] C. Gu , J. S. Lee , ACS Nano 2016, 10, 5413. 27093096 10.1021/acsnano.6b01643

[37] B. Hwang , J. S. Lee , Nanoscale 2018, 10, 8578. 29694471 10.1039/c8nr00863a

[38] M. Lanza , H. S. P. Wong , E. Pop , D. Ielmini , D. Strukov , B. C. Regan , L. Larcher , M. A. Villena , J. J. Yang , L. Goux , A. Belmonte , Y. Yang , F. M. Puglisi , J. Kang , B. Magyari‐Köpe , E. Yalon , A. Kenyon , M. Buckwell , A. Mehonic , A. Shluger , H. Li , T. H. Hou , B. Hudec , D. Akinwande , R. Ge , S. Ambrogio , J. B. Roldan , E. Miranda , J. Suñe , K. L. Pey , X. Wu , N. Raghavan , E. Wu , W. D. Lu , G. Navarro , W. Zhang , H. Wu , R. Li , A. Holleitner , U. Wurstbauer , M. C. Lemme , M. Liu , S. Long , Q. Liu , H. Lv , A. Padovani , P. Pavan , I. Valov , X. Jing , T. Han , K. Zhu , S. Chen , F. Hui , Y. Shi , Adv. Electron. Mater. 2019, 5, 1800143.

[39] L. Song , R. K. Bly , J. N. Wilson , S. Bakbak , J. O. Park , M. Srinivasarao , U. H. F. Bunz , Adv. Mater. 2004, 16, 115.

[40] H. Ling , M. Yi , M. Nagai , L. Xie , L. Wang , B. Hu , W. Huang , Adv. Mater. 2017, 29, 1701333. 10.1002/adma.201701333 28707713

[41] I. Valov , Semicond. Sci. Technol. 2017, 32, 093006.

