# Supplementary material for: Multifunctional Polymer Memory via Bi‐Interfacial Topography for Pressure Perception Recognition
Source: Adv Sci (Weinh). 2020 Feb 25;7(8):1902864. doi: 10.1002/advs.201902864 (PMC7175288; doi:10.1002/advs.201902864)
Supplement: Supplementary file 1 — Supporting Information [file ADVS-7-1902864-s001.pdf]

Copyright WILEY-VCH Verlag GmbH & Co. KGaA, 69469 Weinheim, Germany, 2020.

ADVANCED SCIENCE
Open Access

Supporting Information

for *Adv. Sci.,* DOI: 10.1002/advs.201902864

Multifunctional Polymer Memory via Bi-Interfacial Topography for Pressure Perception Recognition

Xiangjing Wang, Zhe Zhou, Chaoyi Ban, Zepu Zhang, Shang Ju, Xiao Huang, Huiwu Mao, Qing Chang, Yuhang Yin, Mengya Song, Shuai Cheng, Yamei Ding, Zhengdong Liu, Ruolin Ju, Linghai Xie, Feng Miao, Juqing Liu, and Wei Huang**

Supporting Information

Multi-functional polymer memory via bi-interfacial topography for pressure perception recognition

Xiangjing Wang, Zhe Zhou, Chaoyi Ban, Zepu Zhang, Shang Ju, Xiao Huang, Huiwu Mao, Qing Chang, Yuhang Yin, Mengya Song, Shuai Cheng, Yamei Ding, Zhengdong Liu, Ruolin Ju, Linghai Xie, Feng Miao, Juqing Liu, Wei Huang**

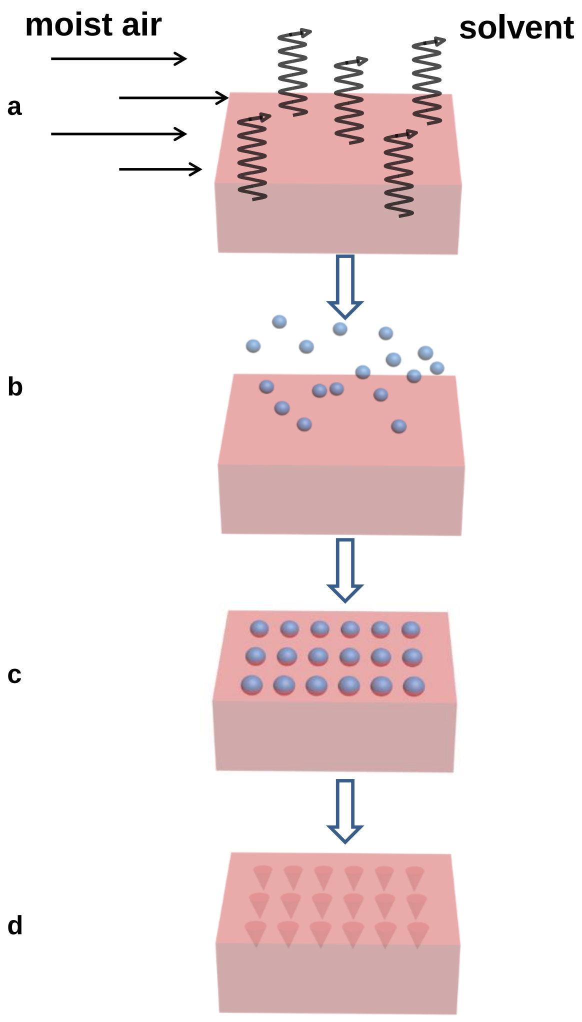

Figure S1. Schematic illustration of a nanohole array formed via the breath figure method is shown. **a**, Moist air results in evaporative cooling of the solvent chloroform. **b**, Water droplets are formed by condensation of the warm moist air onto the cold surface of the liquid. **c**, The water droplets organize into a nanohole array. **d**, The water droplets sink into the solution and, by further evaporation, leave the polymer matrix that is formed by the imprinting of the holes as a fossil.

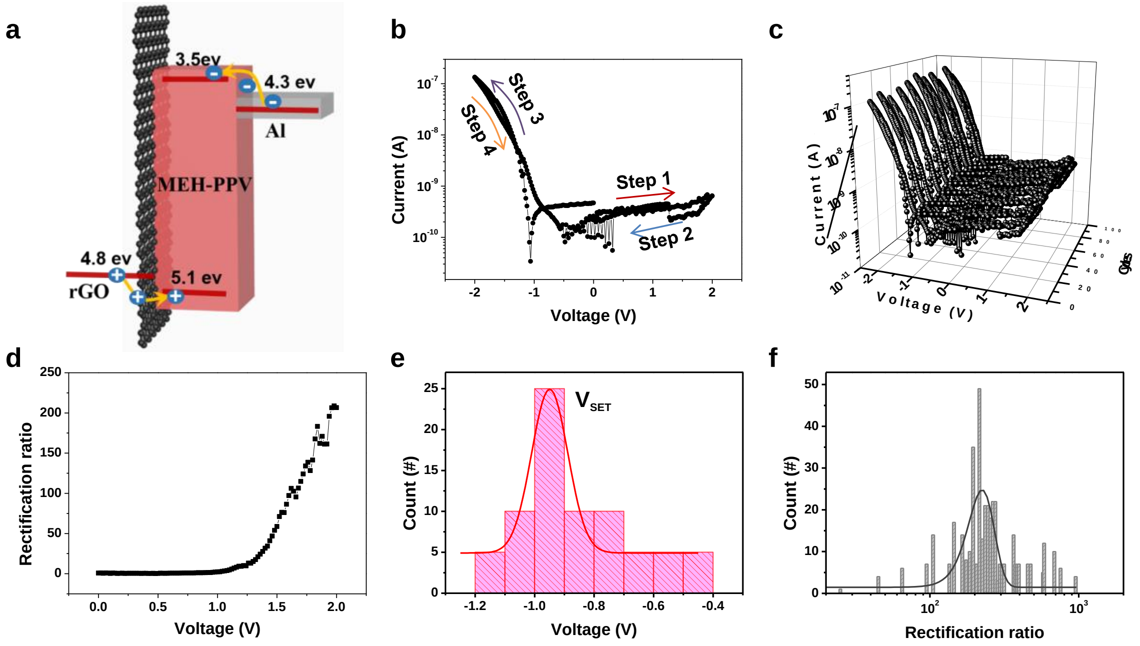

Figure S2. **a**, Energy level diagrams for the multi-functional device is shown. The Schottky barrier for electron diffusion between nh-MEH-PPV/Al and hole diffusion between nw-rGO/nh-MEH-PPV is significantly lower than that for hole diffusion between nh-MEH-PPV/Al and electron diffusion between nw-rGO/nh-MEH-PPV. **b**, In reverse bias, electrons are effectively injected from Al and holes are effectively injected from rGO; whereas in forward bias, both electrons and holes encounter a significant potential barrier. The device exhibits stable rectifying behavior in **c** with a rectifying ratio of ~200 at ±2 V, as shown in **d**. The Gaussian fits of the histograms, as a function of voltage **e** and as a function of rectifying ratio **f**, indicate that there is a set voltage of ~0.95 V and an average rectifying ratio of ~200; it is possible to have a cell-to-cell variation of the devices.

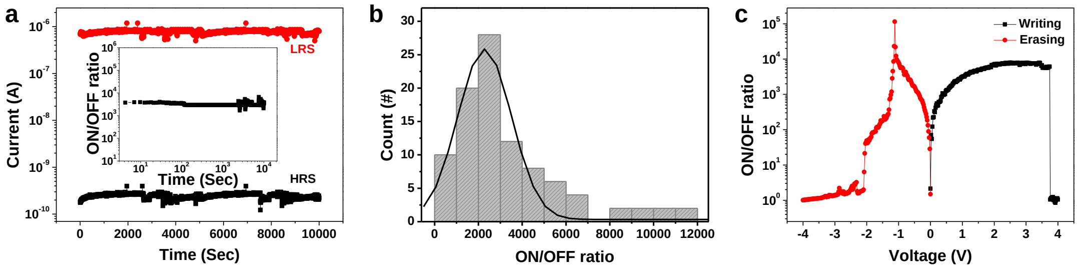

Figure S3. a, Retention performance in both the HRS and the LRS 1 for flash mode (inset: the calculated ON/OFF ratio); **b**, distribution of the ON/OFF ratios obtained for various devices, fitted to a Gaussian function, for flash mode; and **c**, voltage dependence of ON/OFF ratio for flash mode are given.

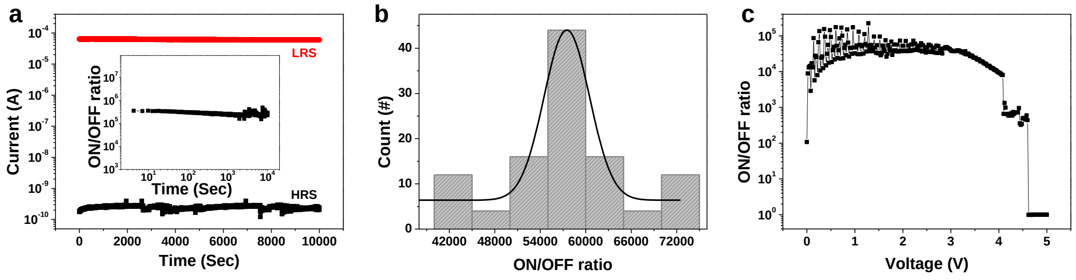

Figure S4. **a**, Retention performance in both the HRS and the LRS 2 for WORM mode (inset: the calculated ON/OFF ratio); **b**, distribution of the ON/OFF ratios obtained for various devices, fitted to a Gaussian function, for WORM mode; and **c**, voltage dependence of ON/OFF ratio for WORM mode are given.

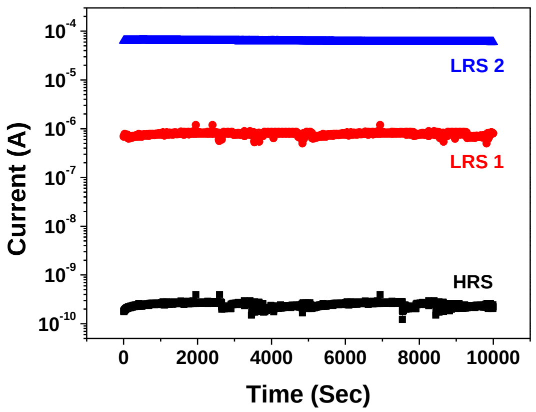

Figure S5. Retention performance of the device utilized for ternary memory.

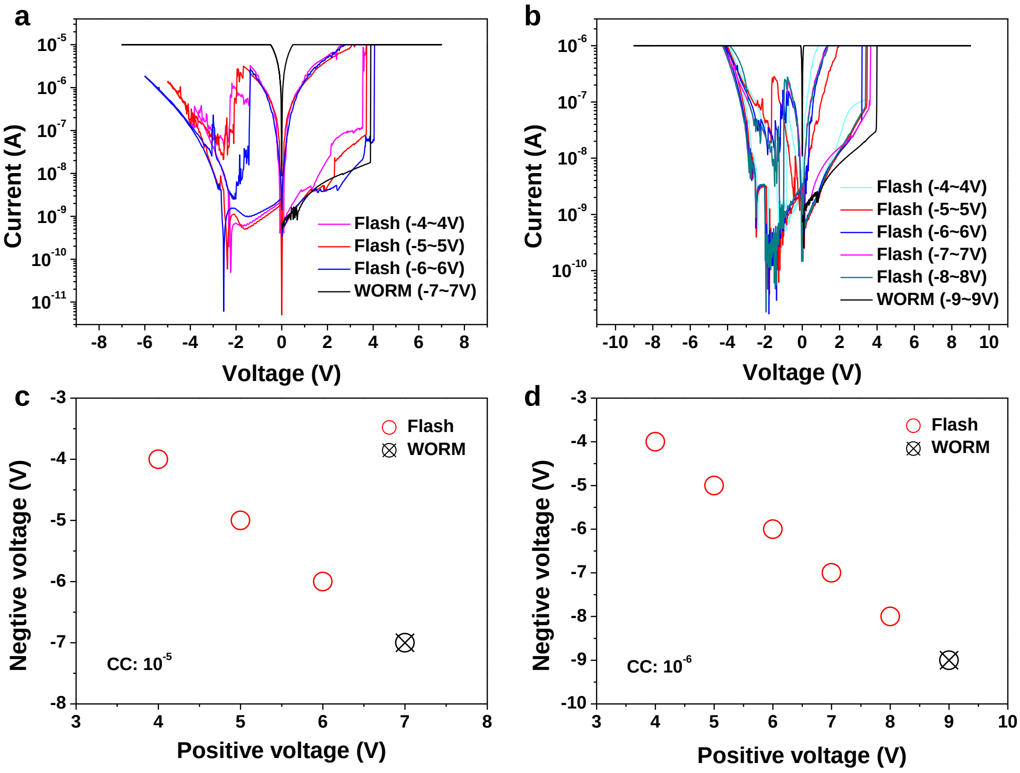

Figure S6. a, Typical *I*-*V* curves for the device undergoing bias -4~4, -5~5, -6~6 and -7~7 V with a CC of 10^{-5} A and **b**, -4~4, -5~5, -6~6, -7~7, -8~8 and -9~9 V with a CC of 10^{-6} A are given. **c** and **d** indicate that the device remains in flash mode at 6 V with a CC of 10^{-5} A and 8 V with a CC of 10^{-6} A, respectively; while the recorded information cannot be erased as the applied bias overcomes 7 V and 9 V, respectively, i.e., the WORM mode.

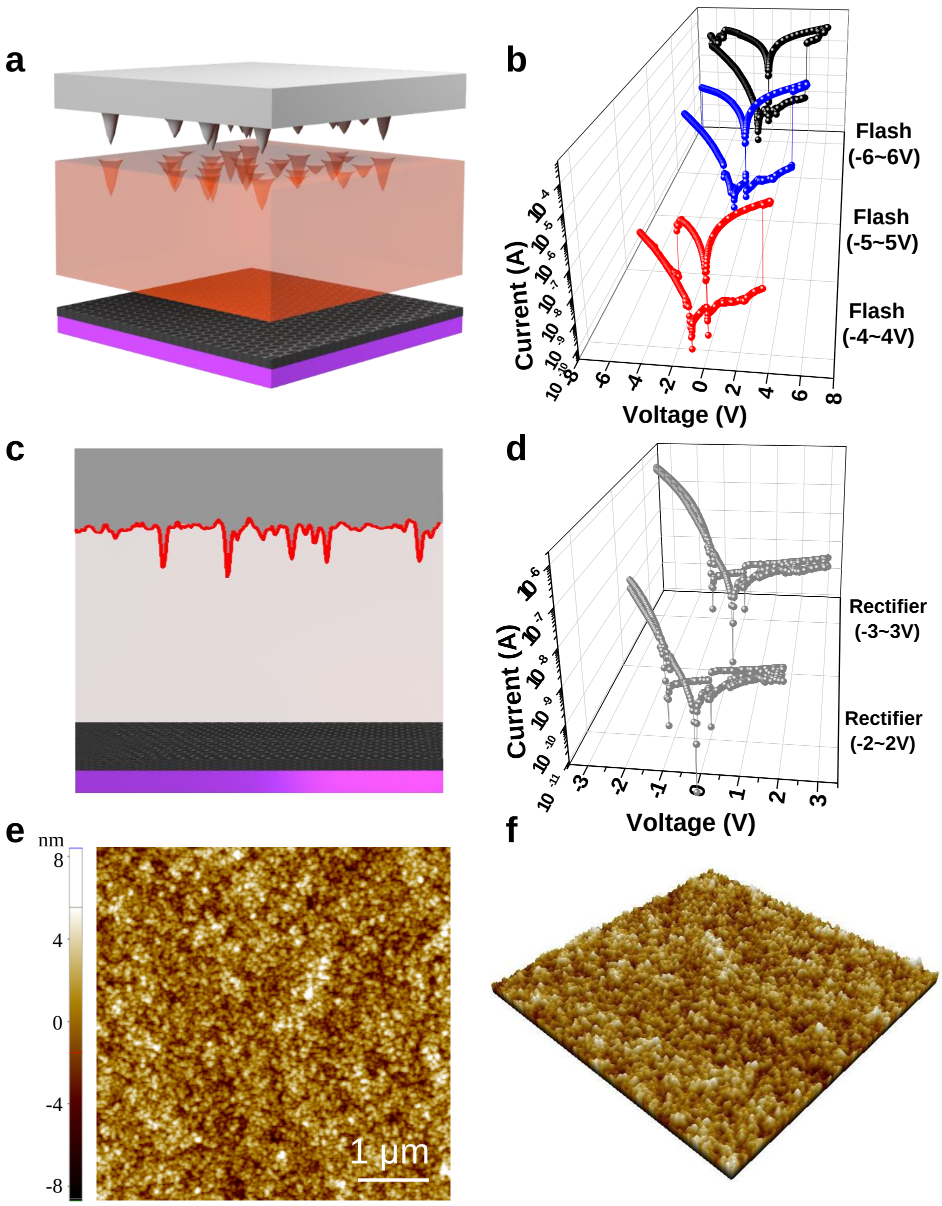

Figure S7. a, Schematic of reference device A with a configuration of s-rGO/nh-MEH-PPV/Al and its profile (**c**). The topographic AFM image (**e** and **f**) indicates a smooth surface for the bottom r-GO electrode with a roughness of ~2.36 nm. The corresponding *I-V* curves show similar rectifying behavior under low bias (-2~2 V and -3~3 V, **d**) yet only flash mode (b) for it underwent identical sweep -4~4, -5~5 and -6~6 V, respectively.

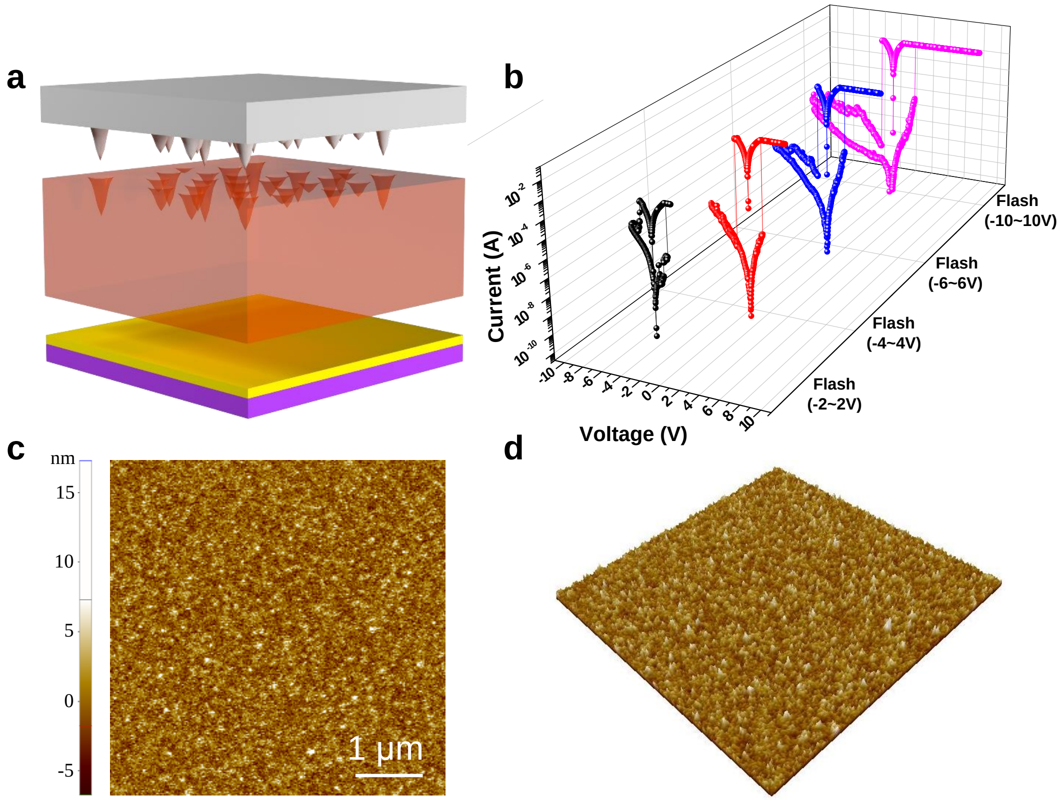

Figure S8. **a**, Schematic of reference device B with a configuration of Au/nh-MEH-PPV/Al is given. **b**, The corresponding *I-V* curves show only flash mode (no WORM mode) for device B for identical sweeps -2~2, -4~4, and -6~6. The topographic AFM images (**c** and **d**) indicate an ultra-smooth surface for the bottom Au electrode with a roughness of ~2.14 nm (bottom). Furthermore, the vanished WORM mode, as the applied bias reached 10 V, suggests that nanowrinkles-induced localized field is essential for the WORM mode.

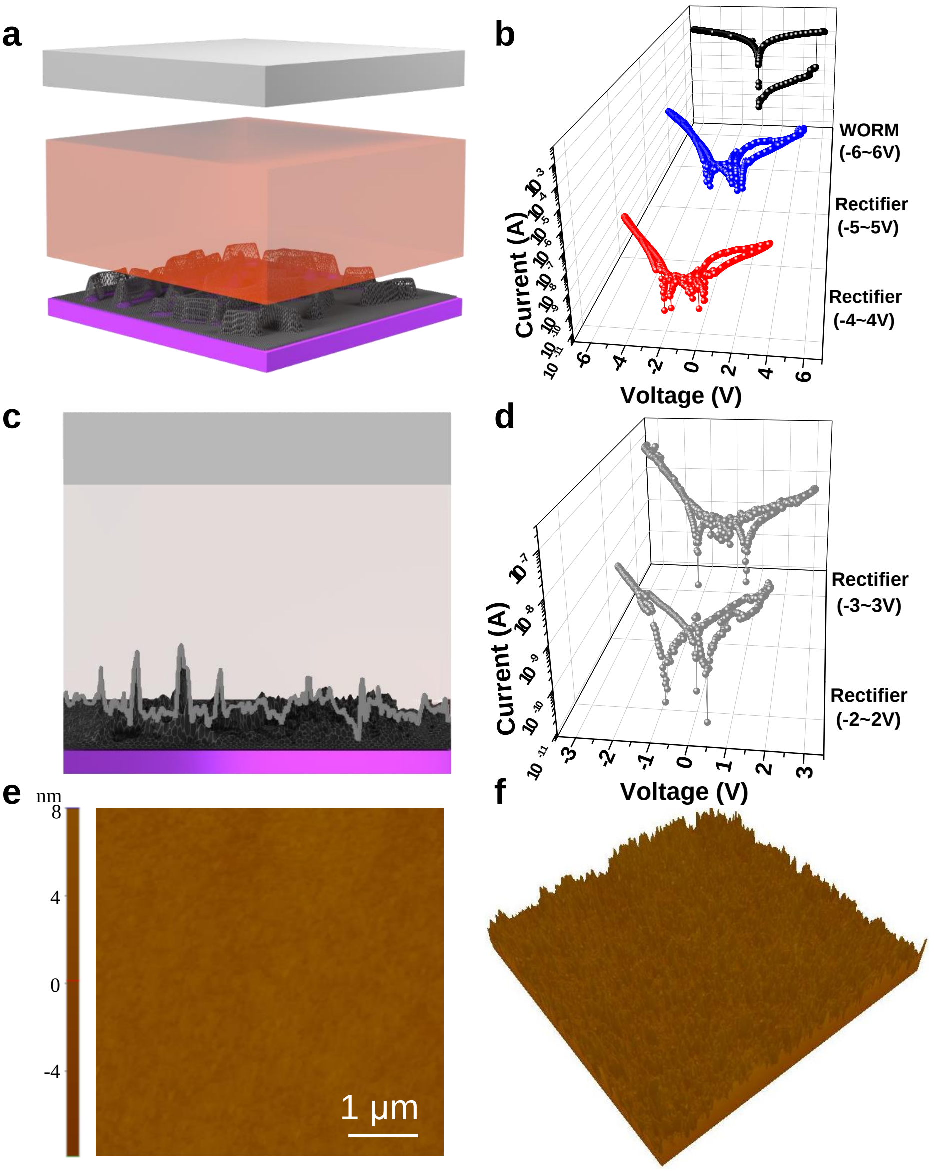

Figure S9. a, **c** Schematic of reference device C with a configuration of nw-rGO/MEH-PPV (without holes)/Al and its profile are given. **b**, There is only WORM mode for identical sweeps of -4~4, -5~5 and -6~6 V. **d**, The corresponding *I-V* curves show similar rectifying behavior under low bias (-2~2 V and -3~3 V). These results indicate that nanoholes are essential for the flash mode. The topographic AFM images (**e** and **f**) indicate a smooth surface for the intervening MEH-PPV film with a roughness of ~2.16 nm.

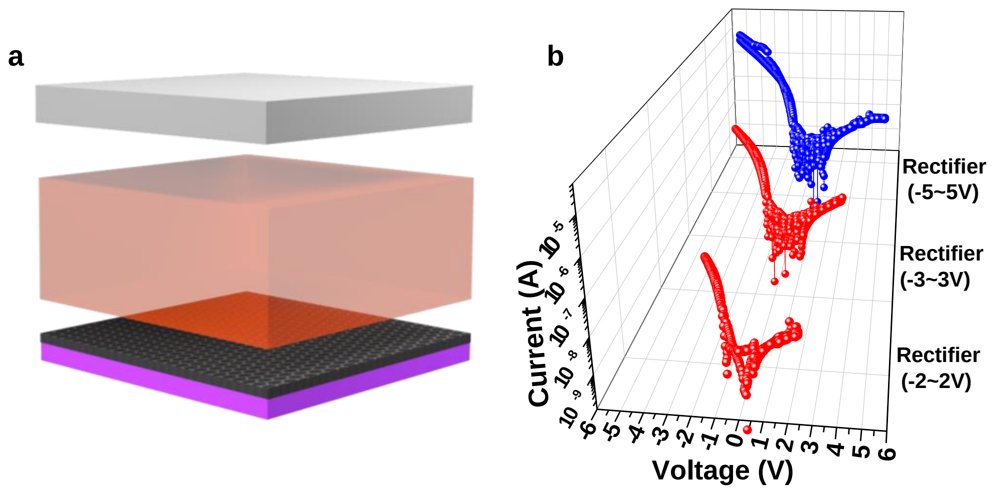

Figure S10. a, Schematic of reference device D with a configuration of s-rGO/MEH-PPV (without holes)/Al is shown. **b**, The corresponding *I*-*V* curves show only rectifying mode; however there is neither flash nor WORM mode for device D for sweeps of -2~2, -3~3 and -5~5 V.

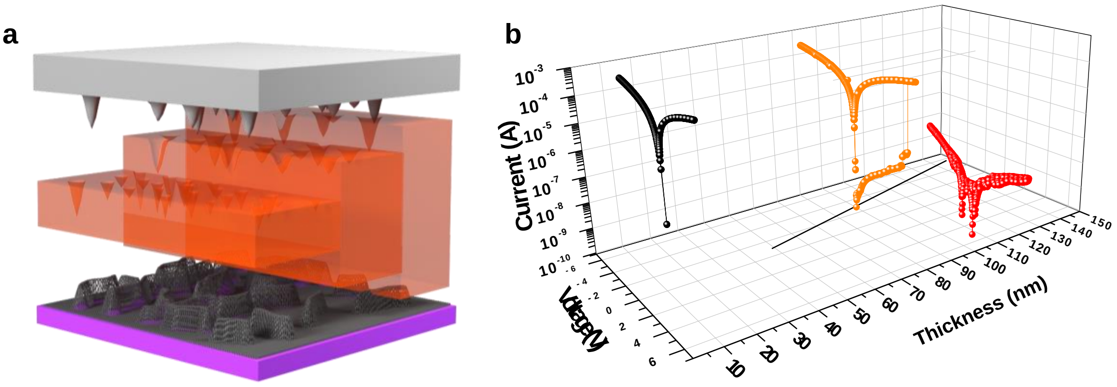

Figure S11. **a**, Schematic of reference devices with different nh-MEH-PPV film thickness is given. **b**, The corresponding *I*-*V* curves show only LRS (black line), WORM (orange line), and HRS (red line) for the nh-MEH-PPV film thickness of 10, 80, and 150 nm, respectively; this indicates that an appropriate thickness is crucial for the multimode switching performance of the device.

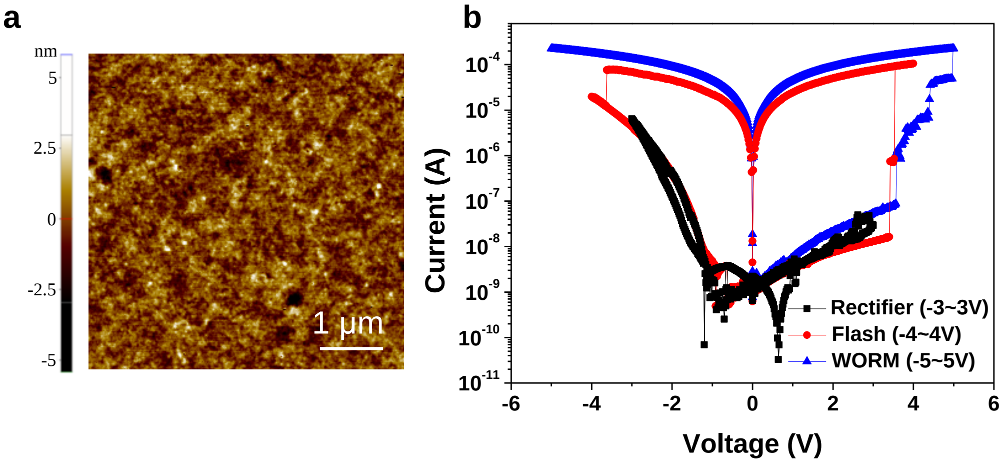

Figure S12. AFM image of P3HT film (**a**) with nanohole structures and the corresponding *I*-*V* curves (b) are given. Multi-mode switching behaviors (red line for flash mode and blue line for WORM mode) can be observed.

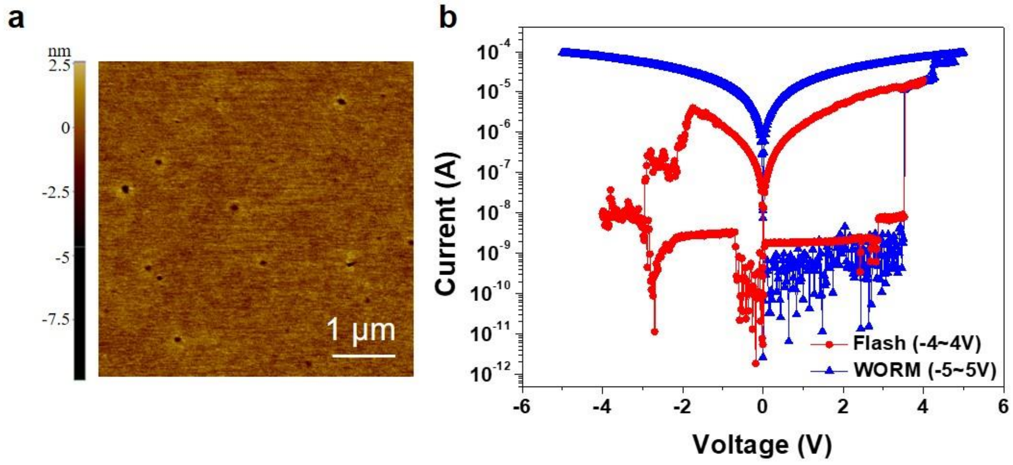

Figure S13. AFM image of PMMA film (**a**) with nanoholes and the corresponding *I*-*V* curves (**b**) are given. Multi-mode switching behavior (red line for flash mode and blue line for WORM mode) can be observed.

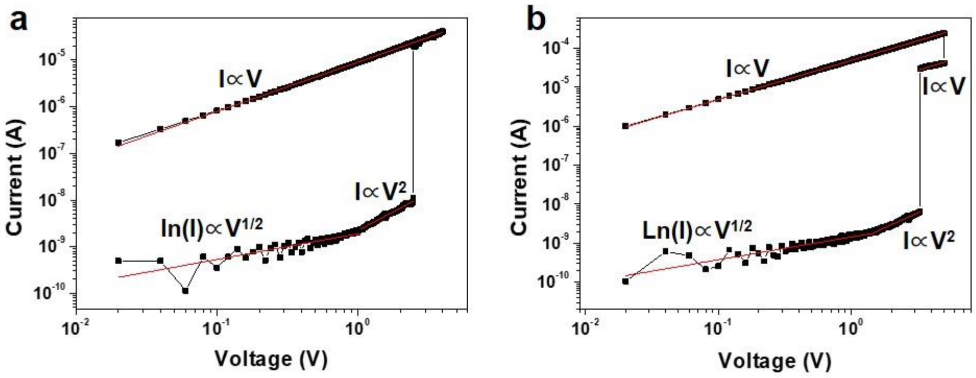

Figure S14. **a**, *ln I-ln V* relationship for the forward bias regime of flash mode is given. **b**, The fitting results indicate that thermionic emission (*ln(V)* vs. $V^{1/2}$, blue line in top right) and space-charge limited conduction (SCLC, $I \propto V^2$, black line in top right) dominate electrical conduction in the HRS and there is Ohmic conduction in the LRS 1 (black line in top right).

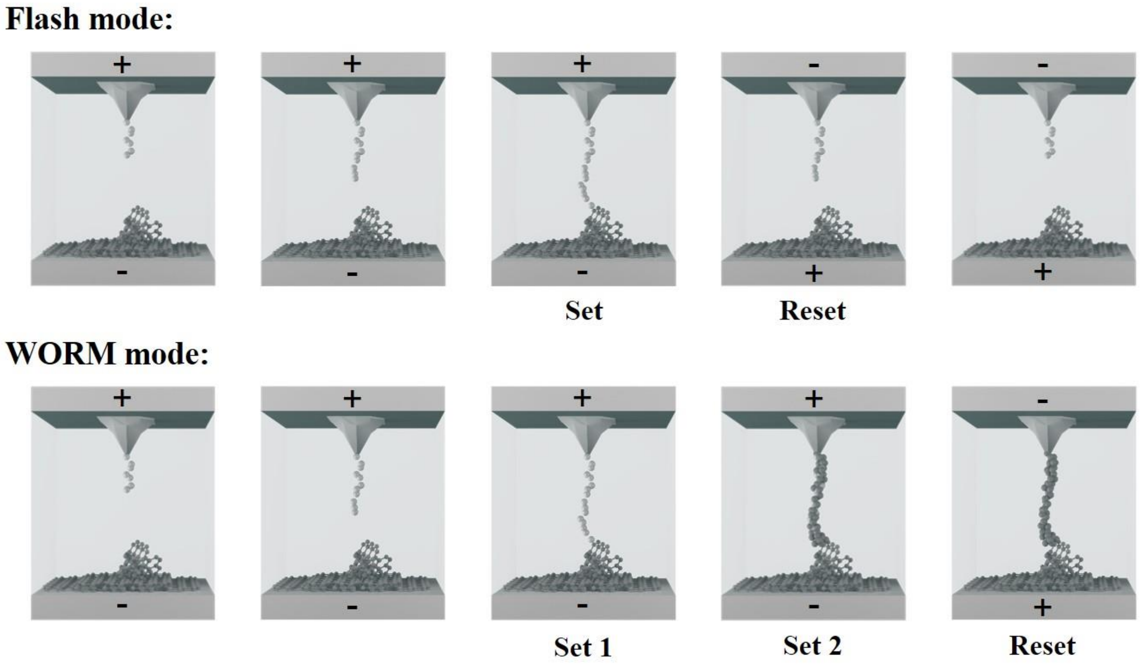

Figure S15. Schematic illustration to propose a resistive switching model for the multi-functional device. In the case of flash mode, the switching behavior is ascribed to the formation and rupture of the Al filaments. In the case of WORM mode, the switching behavior results from the localized electrical field-induced formation of carbon-rich filaments.
